# Atrial fibrillation in patients undergoing coronary artery surgery is associated with adverse outcome

**DOI:** 10.1080/03009734.2018.1504148

**Published:** 2018-09-28

**Authors:** Gorav Batra, Anders Ahlsson, Bertil Lindahl, Lars Lindhagen, Anders Wickbom, Jonas Oldgren

**Affiliations:** a Uppsala Clinical Research Center, Uppsala, Sweden;; b Department of Medical Sciences, Cardiology, Uppsala University, Uppsala, Sweden;; c Department of Cardiothoracic and Vascular Surgery, School of Medicine and Health, Örebro University, Örebro, Sweden

**Keywords:** Atrial fibrillation, cardiovascular disease, coronary artery bypass grafting

## Abstract

**Background:** The aim was to determine the association between atrial fibrillation (AF) and outcome in patients undergoing coronary artery bypass grafting (CABG).

**Methods:** All patients undergoing CABG between January 2010 and June 2013 were identified in the Swedish Heart Surgery Registry. Outcomes studied were all-cause mortality, cardiovascular mortality, myocardial infarction, congestive heart failure, ischemic stroke, and recurrent AF. Patients with history of AF prior to surgery (preoperative AF) and patients without history of AF but with AF episodes post-surgery (postoperative AF) were compared to patients with no AF using adjusted Cox regression models.

**Results:** Among 9,107 identified patients, 8.1% (*n* = 737) had preoperative AF, and 25.1% (*n* = 2,290) had postoperative AF. Median follow-up was 2.2 years. Compared to no AF, preoperative AF was associated with higher risk of all-cause mortality, adjusted hazard ratio with 95% confidence interval (HR) 1.76 (1.33–2.33); cardiovascular mortality, HR 2.43 (1.68–3.50); and congestive heart failure, HR 2.21 (1.72–2.84). Postoperative AF was associated with risk of all-cause mortality, HR 1.27 (1.01–1.60); cardiovascular mortality, HR 1.52 (1.10–2.11); congestive heart failure, HR 1.47 (1.18–1.83); and recurrent AF, HR 4.38 (2.46–7.78). No significant association was observed between pre- or postoperative AF and risk for myocardial infarction and ischemic stroke.

**Conclusions:** Approximately 1 in 3 patients undergoing CABG had pre- or postoperative AF. Patients with pre- or postoperative AF were at higher risk of all-cause mortality, cardiovascular mortality, and congestive heart failure, but not of myocardial infarction or ischemic stroke. Postoperative AF was associated with higher risk of recurrent AF.

## Introduction

Atrial fibrillation (AF) is a common arrhythmia with an increasing incidence due to aging population (1). Patients with AF have an increased risk of mortality and morbidity, including ischemic stroke (2,3). History of preoperative AF is a common finding among patients undergoing coronary artery bypass grafting (CABG), with prevalence varying between 7% and 9% (4,5). To our knowledge, only two single-center studies have reported outcome in patients with preoperative AF undergoing CABG, both reporting an increased risk of long-term mortality (4,5). Moreover, previous studies have reported that postoperative AF may occur in approximately one-third of patients undergoing CABG (6,7). To date, most studies on patients undergoing CABG have studied the effects of postoperative AF on mortality and stroke (6–11). Previous studies have found postoperative AF to be associated with increased risk of short-term mortality and stroke (6,8,9). Also, some data suggest that postoperative AF might be associated with long-term mortality (7, 9–11). However, only one small single-center study has simultaneously evaluated both pre- and postoperative AF in relation to outcome in patients undergoing CABG (4). In addition, only two single-center studies have assessed the risk of recurrent AF in patients with new-onset postoperative AF, with data suggesting an increased risk (7,12).

In this study on patients undergoing CABG, based on data from Swedish registers with complete national coverage, we sought to answer the following question: In patients undergoing isolated CABG, what are the impacts of preoperative AF and new-onset postoperative AF on all-cause and cardiovascular mortality, myocardial infarction, congestive heart failure, ischemic stroke, and recurrent AF?

## Materials and methods

### Study population and data collection

Clinical and procedural data were obtained from the Swedish Web-System for Enhancement and Development of Evidence-Based Care in Heart Disease Evaluated According to Recommended Therapies (SWEDEHEART) registry and was enriched with data from the National Patient Registry and the National Dispensed Drug Registry. SWEDEHEART is a national quality registry that contains several sub-registries, including the Swedish Heart Surgery Registry which captures data about all patients undergoing thoracic surgery in Sweden. The SWEDEHEART registry also includes data about all patients admitted to coronary care units due to symptoms indicative of acute coronary syndrome and data on patients undergoing coronary angiography (13). Patients included in SWEDEHEART are informed about their participation in the registry but do not provide written consent; however, they have the right to withdraw their participation. Mortality data were obtained from the Swedish Cause of Death registry, which is a mandatory nationwide registry that has collected vital status of all Swedish citizens since 1961. The National Patient Registry is a mandatory nationwide registry that includes discharge diagnosis for all patients admitted to Swedish hospitals since 1987. Previous studies have shown that the registry has high validity for several diagnoses, including myocardial infarction, congestive heart failure, and stroke (14). The National Dispensed Drug Registry has captured data about all prescribed drugs dispensed at Swedish pharmacies since 2005 (15). In our study, baseline pharmacologic treatment was determined based on drugs dispensed 6 months prior to admission and within 30 days from surgery. Data linkage between registries was approved and performed by the National Board of Health and Welfare in Sweden using the unique 10-digit identifier relating to all Swedish citizens. See Supplementary Table 1 (available online) for detailed description of data collection, linkage, and variable definition. The ethics committee at Karolinska Institute, Stockholm approved the study protocol.

**Figure 1.**
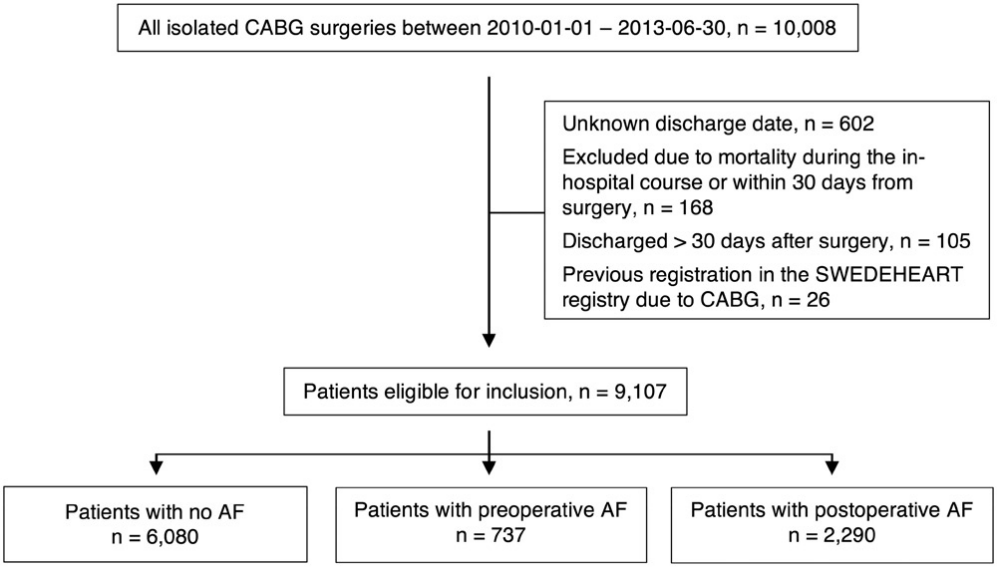
Study population.

In the present study, inclusion period was defined between January 2010 and June 2013. During this period, 10,008 consecutive patients underwent isolated CABG at eight sites and were identified in SWEDEHEART. Among identified patients, 9,107 individuals were eligible for inclusion. Patients were excluded if they died within 30 days from surgery, had missing discharge date, were discharged over 30 days after surgery, or had a previous registration in SWEDEHEART due to CABG (Figure 1).

### Definition of exposure

The main exposure was AF. History of AF, i.e. preoperative AF, was considered to be present if indicated as occurring before surgery in SWEDEHEART, or if the diagnosis of AF appeared in the National Patient Registry prior to admission. New-onset postoperative AF was considered in patients with no history of AF according to SWEDEHEART and the National Patient Registry, but with an episode of AF post-surgery according to SWEDEHEART. As controls, we used patients with no diagnosis of AF pre- or post-CABG.

### Definition of outcome

Outcomes under investigation were all-cause mortality, cardiovascular mortality, myocardial infarction, hospitalization due to congestive heart failure, ischemic stroke, and recurrent AF. Outcomes were identified in the National Patient Registry and in the National Cause of Death Registry using International Classification of Diseases 10th revision codes; see Supplementary Table 2 (available online) for details. For recurrent AF, direct-current (DC) cardioversion was used as a surrogate marker of symptomatic relapse in AF as previously reported (16). As a sensitivity analysis, diagnosis of recurrent AF in the National Patient Registry was accounted for, however, with the risk of AF diagnosis codes being used for both follow-up visits and for true relapses. Time at risk was counted from 30 days after CABG surgery. This blanking period was applied to avoid registration of in-hospital complications and double-counting of events when patients moved between hospital wards. When analyzing outcome, complete follow-up was available for all cases. Patients were censored at the end of follow-up, which lasted up to 31 December 2013, giving a minimum follow-up of 6 months. For all-cause mortality, no other censoring scheme was applied. For cardiovascular mortality, myocardial infarction, congestive heart failure, ischemic stroke, and recurrent diagnosis of AF, patients were also censored for all-cause mortality.

### Statistical analyses

Demographics, baseline characteristics, EuroSCORE I scoring system for prediction of mortality in patients undergoing cardiac surgery (17), hospital course, perioperative data, and discharge medication among patients with no AF, preoperative AF, and postoperative AF were presented descriptively using percentages for categorical variables and with median and interquartile range for continuous variables. To test for differences, Pearson’s chi-square test was used for categorical variables and the Kruskal–Wallis test for continuous variables. The risk of all-cause mortality and recurrent diagnosis of AF in relation to pre- and postoperative AF was illustrated using Kaplan–Meier survival plots. Event rates according to the number of events per 100 person-years were calculated. Unadjusted and adjusted Cox proportional-hazards regression models were estimated for each outcome and presented using hazard ratios (HR) with 95% confidence interval (CI). In the unadjusted models, AF status was entered as the sole variable. In the adjusted models, factors in the CHA_2_DS_2_-VASc scoring system (congestive heart failure, hypertension, age [3 knot restricted cubic spline], diabetes mellitus, stroke, transient ischemic attack or thromboembolism, vascular disease, and sex) (18), hospital (Γ distributed random frailty effect), and year of inclusion were accounted for. No data were missing in regard to the variables included in the adjusted Cox proportional-hazards regression models.

As sensitivity analysis, a propensity-score matched analysis was performed in which propensity scores for the likelihood of no AF or postoperative AF were obtained using random effects logistic regression models with all variables in the adjusted Cox proportional-hazards regression models as explanatory variables. Matching was done in 1:1 based on estimated propensity scores with a caliper of 0.001, resulting in 2,211 patients in the no AF cohort and 2,211 patients in the postoperative AF cohort (19).

All statistical analyses were conducted at Uppsala Clinical Research Center using R version 3.1.0. All statistical tests were two-sided using a *p* values of <0.05 as significant.

## Results

### Study population and patient characteristics

The study population had a median age of 68 years, and 18.9% were women. A history of AF prior to CABG was documented in 737 (8.1%) patients, and 2,290 (25.1%) patients had new-onset postoperative AF. Table 1 summarizes patient characteristics stratified by AF status. Patients with preoperative AF were older than patients with postoperative AF, and accordingly more likely to have concomitant diseases. Compared to patients with no AF, patients with postoperative AF were older and more likely to have comorbidities such as hypertension, history of myocardial infarction, congestive heart failure, and chronic obstructive pulmonary disease. Compared to patients with no AF, a higher proportion of patients with pre- and postoperative AF had reduced left ventricular ejection fraction and higher creatinine values. Likewise, the EuroSCORE 1 was numerically higher among patients with pre- and postoperative AF. Additional perioperative and postoperative data are presented in Table 1. At discharge, patients with preoperative AF versus postoperative AF were more likely to receive oral anticoagulants, while patients with postoperative AF were more likely to receive aspirin and/or P2Y_12_ inhibitors. However, compared to patients with no AF, patients with postoperative AF were more likely to receive oral anticoagulants, diuretics, digoxin, sotalol, and amiodarone at discharge.

**Table 1.** Baseline table: patient characteristics, admission year, and clinical and in-hospital characteristics of coronary artery bypass graft surgery patients in relation to atrial fibrillation status.

Variable	No AF (*n* = 6,080)	Preoperative AF (*n* = 737)	Postoperative AF (*n* = 2,290)	*P* value
Demographics				
Age, median (IQR), years	66 (60–72)	72 (67–77)	70 (64–75)	<0.001
Sex, women	1,171 (19.3)	141 (19.1)	406 (17.7)	0.27
Smoking, *n* = 8,493	1,031 (18.3)	84 (12.2)	302 (14.0)	<0.001
BMI, median (IQR), *n* = 8,990	27.1 (24.7–29.7)	27.2 (24.6–30.1)	27.0 (24.7–29.7)	0.55
Admission year				0.009
2010	1,901 (31.3)	202 (27.4)	643 (28.1)	
2011	1,762 (29.0)	206 (28.0)	660 (28.8)	
2012	1,670 (27.5)	223 (30.3)	657 (28.7)	
2013	747 (12.3)	106 (14.4)	330 (14.4)	
Comorbidities and presentation at admission				
Diabetes mellitus	1,851 (30.4)	285 (38.7)	675 (29.5)	<0.001
Hypertension	4,128 (67.9)	594 (80.6)	1,697 (74.1)	<0.001
Myocardial infarction	3,595 (59.1)	560 (76.0)	1,421 (62.1)	<0.001
Congestive heart failure	974 (16.0)	280 (38.0)	406 (17.7)	<0.001
Peripheral vascular disease	264 (4.3)	56 (7.6)	110 (4.8)	<0.001
Thromboembolism	14 (0.2)	3 (0.4)	10 (0.4)	0.26
Ischemic / unknown stroke	287 (4.7)	60 (8.1)	114 (5.0)	<0.001
Transient ischemic attack	115 (1.9)	37 (5.0)	69 (3.0)	<0.001
Hemorrhagic stroke	44 (0.7)	4 (0.5)	15 (0.7)	0.83
Any bleeding	291 (4.8)	63 (8.5)	98 (4.3)	<0.001
Renal failure	125 (2.1)	50 (6.8)	56 (2.4)	<0.001
Chronic obstructive pulmonary disease	319 (5.2)	62 (8.4)	138 (6.0)	0.002
Cancer diagnosis within 3 years	146 (2.4)	33 (4.5)	61 (2.7)	0.004
Previous PCI	1,365 (22.5)	184 (25.0)	490 (21.4)	0.13
Previous CABG	99 (1.6)	21 (2.8)	35 (1.5)	0.04
Hospital course and perioperative data				
Indication for CABG, *n* = 8,886				<0.001
Stable coronary artery disease	2,533 (42.8)	232 (32.4)	921 (41.0)	
Unstable angina / Acute MI	3,389 (57.2)	485 (67.6)	1,326 (59.0)	
EuroSCORE I, median (IQR), *n* = 9,062	4 (2–6)	6 (4–8)	5 (3–7)	<0.001
Extracorporeal circulation	6,014 (98.9)	724 (98.2)	2,256 (98.5)	0.14
Number of central anastomoses, *n* = 7,434				<0.001
0	413 (8.5)	48 (8.4)	96 (4.8)	
1	2,153 (44.1)	254 (44.4)	876 (44.2)	
2	2,163 (44.3)	250 (43.7)	938 (47.3)	
≥3	149 (3.1)	20 (3.5)	74 (3.7)	
Number of peripheral anastomoses, *n* = 7,434				<0.001
0	250 (5.1)	17 (3.0)	33 (1.7)	
1	149 (3.1)	24 (4.2)	59 (3.0)	
2	919 (18.8)	106 (18.5)	372 (18.8)	
≥3	3,560 (73.0)	425 (74.3)	1,520 (76.6)	
Internal mammary artery, left, *n* = 7,434	4,352 (89.2)	497 (86.9)	1,834 (92.4)	<0.001
Internal mammary artery, right, *n* = 7,434	92 (1.9)	6 (1.0)	30 (1.5)	0.24
Vein graft, *n* = 7,434	4,465 (91.5)	525 (91.8)	1,882 (94.9)	<0.001
Radial artery graft, *n* = 7,434	59 (1.2)	15 (2.6)	49 (2.5)	<0.001
Postoperative bleeds, *n* = 9,080	207 (3.4)	31 (4.2)	113 (4.9)	0.005
Postoperative stroke, *n* = 9,093	48 (0.8)	12 (1.6)	35 (1.5)	0.003
Left ventricular ejection fraction <50%, *n* = 8,160	1,466 (27.0)	307 (44.7)	609 (29.7)	<0.001
Creatinine, median (IQR), µmol/L, *n* = 9,100	87 (75–105)	100 (83–129)	93 (79–120)	<0.001
CHA_2_DS_2_-VASc score at discharge				<0.001
CHA_2_DS_2_-VASc score =0	0 (0.0)	0 (0.0)	0 (0.0)	
CHA_2_DS_2_-VASc score =1	638 (10.5)	13 (1.8)	113 (4.9)	
CHA_2_DS_2_-VASc score ≥2	5,442 (89.5)	724 (98.2)	2,177 (95.1)	
Discharge medication				
Aspirin	5,539 (91.1)	512 (69.5)	1,958 (85.5)	<0.001
P2Y_12_ inhibitors	955 (15.7)	61 (8.3)	311 (13.6)	0.52
Oral anticoagulants	278 (4.6)	338 (45.9)	417 (18.2)	<0.001
ACEI/ARB	4,158 (68.4)	522 (70.8)	1,558 (68.0)	<0.001
Calcium channel blockers	1,146 (18.8)	158 (21.4)	473 (20.7)	<0.001
Diuretics	2,155 (35.4)	402 (54.5)	1,075 (46.9)	<0.001
Statins	5,492 (90.3)	617 (83.7)	2,034 (88.8)	0.54
Digoxin	37 (0.6)	89 (12.1)	44 (1.9)	<0.001
β-blockers	5,433 (89.4)	629 (85.3)	1,973 (86.2)	<0.001
Sotalol	39 (0.6)	27 (3.7)	229 (10.0)	<0.001
Amiodarone	127 (2.1)	111 (15.1)	417 (18.2)	<0.001
Verapamil / diltiazem	59 (1.0)	16 (2.2)	30 (1.3)	<0.001

Characteristics were reported using percentages for categorical variables, or with median and IQR for continuous variables (as noted).

ACEI = angiotensin-converting enzyme inhibitors; AF = atrial fibrillation; ARB = angiotensin II receptor blockers; BMI = body mass index; CABG = coronary artery bypass grafting; CHA_2_DS_2_-VASc = congestive heart failure, hypertension, age ≥75 years, diabetes mellitus, prior stroke, transient ischemic attack or thromboembolism, vascular disease, age 65–74 years, female sex; IQR = interquartile range; PCI = percutaneous coronary intervention.

### Outcome analyses

**Figure 2.**
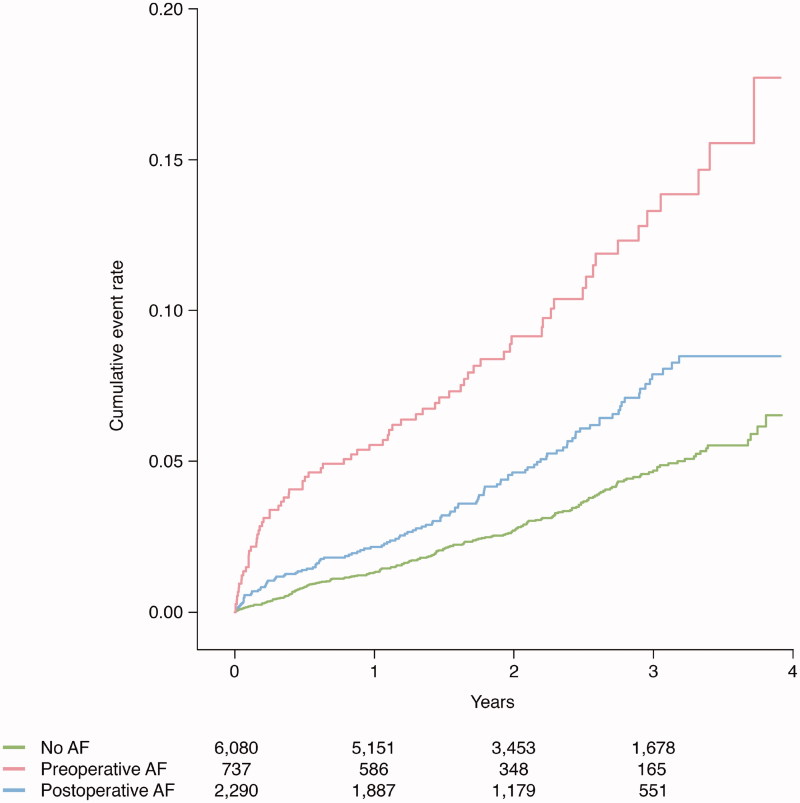
Event rate of all-cause mortality in relation to atrial fibrillation status.

**Figure 3.**
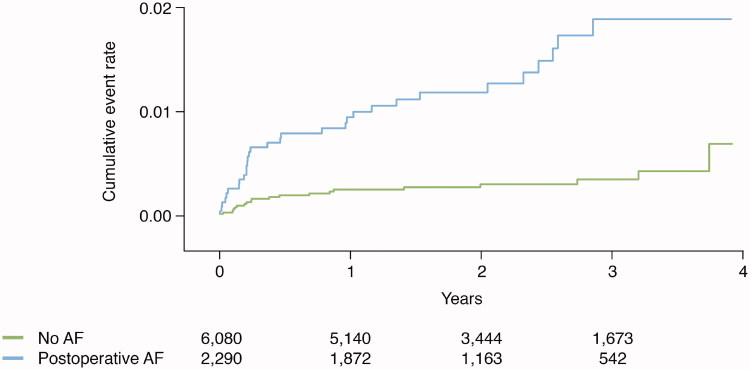
Event rate of recurrent symptomatic atrial fibrillation in relation to atrial fibrillation status.

Clinical outcomes post-CABG were evaluated according to preoperative AF versus no AF and postoperative AF versus no AF. Median follow-up was 2.2 years. Figures 2 and 3 present unadjusted Kaplan–Meier plots illustrating cumulative event rates for all-cause mortality and recurrent AF within 4 years of post-surgery, with exclusion of a 30-day blanking period, stratified by AF status at baseline.

**Figure 4.**
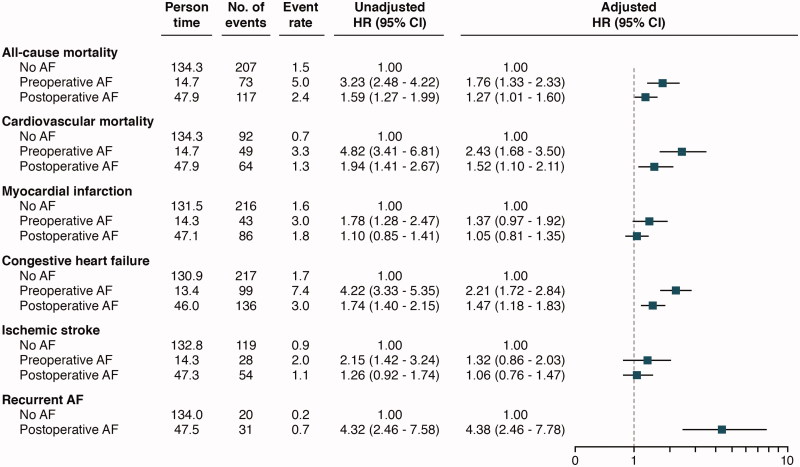
Event rates according to the number of events per 100 person-years. Unadjusted and adjusted hazard ratios with a 95% confidence interval in relation to atrial fibrillation status, with no atrial fibrillation as reference.

Unadjusted cumulative event rates per 100 person-years for all-cause mortality and cardiovascular mortality were numerically higher in patients with pre- and postoperative AF compared to no AF. Using Cox proportional-hazards regression models with no AF as reference, and with adjustment for clinically relevant variables, preoperative AF and postoperative AF were associated with higher risk of all-cause mortality, adjusted hazard ratio (HR) with 95% confidence interval 1.76 (1.33–2.33) and HR 1.27 (1.01–1.60), respectively (Figures 2 and 4). For cardiovascular mortality, a similar risk was observed for patients with pre- and postoperative AF, HR 2.43 (1.68–3.50) and HR 1.52 (1.10–2.11), respectively. As compared to no AF, patients with pre- and postoperative AF had a higher number of hospitalizations due to congestive heart failure during follow-up, with similar findings found in adjusted analyses, HR 2.21 (1.72–2.84) and HR 1.47 (1.18–1.83), respectively. In patients with preoperative AF versus no AF, a higher non-adjusted HR was recorded in regard to myocardial infarction, but with a non-significant adjusted HR of 1.37 (0.97–1.92). For postoperative AF versus no AF, no significant observation was made in regard to myocardial infarction. Preoperative AF and postoperative AF were not associated with ischemic stroke (Figure 4).

The cumulative incidence rate per 100 person-years for recurrent symptomatic AF after discharge was numerically higher for patients with new-onset postoperative AF compared with no AF (0.7 versus 0.2). This association persisted after adjustments; HR 4.38 (2.46–7.78) (Figures 3 and 4).

### Sensitivity analyses

Two sensitivity analyses were conducted. First, risk of relapse in AF was analyzed in a model in which all recurrent diagnoses of AF in the National Patient Registry, not only DC cardioversion, were accounted for. In the analysis, similar increased risk for recurrent AF as in the main analysis was observed among patients with postoperative AF, HR 3.60 (2.99–4.35). Second, propensity score analyses were performed for all outcomes. In such analyses, similar results as in the main analyses were observed between postoperative AF versus no AF in regard to all outcomes; see Supplementary Table 3 (available online).

## Discussion

In this nationwide cohort of 9,107 consecutive patients undergoing isolated CABG, 8.1% of the patients had a preoperative history of AF prior to surgery, and new-onset postoperative AF complicated 25.1% of the patients. Pre- and postoperative AF were both significantly associated with higher risk of long-term all-cause mortality, cardiovascular mortality, and hospitalization due to congestive heart failure. However, no significant association was observed between AF status and risk for ischemic stroke and myocardial infarction. Compared with no AF, postoperative AF was associated with a higher risk of recurrent symptomatic AF after discharge.

To our knowledge, this study is the first and only large multicenter study with complete coverage to compare several long-term outcomes, including all-cause mortality, cardiovascular mortality, myocardial infarction, congestive heart failure, and ischemic stroke, in patients with pre- and postoperative AF after CABG. Moreover, this is the only nationwide study to assess the long-term risk of recurrent AF after CABG in patients with postoperative AF. Further, we believe this is the only multicenter study to compare different subtypes of AF (pre- and postoperative AF) versus no AF, which has previously only been done in one small single-center study (4). Several studies, preceding ours, have shown an association between postoperative AF and long-term mortality (7,9–11), e.g. in an Australian multicenter study based on 19,497 patients undergoing isolated CABG where postoperative AF was associated with a higher long-term risk of all-cause mortality (odds ratio [OR] 1.19 [1.06–1.32]) (10), as well as a US single-center study based on 16,169 patients undergoing isolated CABG (HR 1.21 [1.12–1.32]) (11), but a slightly higher mortality rate was reported in a prior small Swedish single-center study based on 571 patients undergoing CABG (HR 1.57 [1.05–2.34]) (7). In our study we also found a strong association between postoperative AF and cardiovascular mortality. In addition, we found that 8.1% of our CABG population had a history of AF preceding surgery which was significantly associated with an increased long-term risk of all-cause mortality and cardiovascular mortality. Despite an increased risk of cardiovascular mortality, no association was seen in regard to myocardial infarction for patients with postoperative AF, while patients with preoperative AF had a non-significant 37% higher risk of myocardial infarction. This leads to the notion that other causes of cardiovascular mortality might have an influence, such as the substantially higher risk of congestive heart failure among patients with pre- and postoperative AF as observed in our study. Several other speculative mechanisms for the association of AF and mortality have been proposed in the literature. These include AF recurrence leading to hemodynamic instability (20), and negative proarrhythmic effects due to higher prescription of antiarrhythmic medication among patients with AF (21,22). However, these theories are hypothetical, and to our knowledge no biological and pathophysiological studies are available clarifying casual pathways.

Another important finding in our study was that postoperative AF predicts recurrent AF. During a median follow-up of 2.2 years, patients with postoperative AF had a more than 4-times higher risk of relapse in symptomatic AF as compared to patients with no AF at baseline. These results are similar to the above-mentioned Swedish single-center study, in which the authors reported an increased risk of development of recurrent AF in patients with postoperative AF (OR 8.31 [4.20–16.43]) (7). Also, similar findings with increased risk of recurrent AF have been reported in a Korean single-center study based on 1,171 patients undergoing CABG (HR 5.25 [1.75–15.77]) (12). Despite the high recurrence rate of AF in our study, we were not able to show a significant increased risk of ischemic stroke during follow-up. To our knowledge, only two large studies have explored the relationship between postoperative AF and long-term risk of ischemic stroke. In a US multicenter study based on 73,543 patients undergoing any cardiac surgery, postoperative AF was associated with higher risk of ischemic stroke during long-term follow-up (HR 1.3 [1.1–1.6]) (23). Similar estimates were shown in a Canadian single-center study based on 8,058 patients undergoing isolated CABG (HR 1.26 [1.08–1.47]) (24). The differences in results between our study and the two above-mentioned studies might be explained by the number of patients receiving oral anticoagulants at discharge. In our study, 18.2% of the patients with postoperative AF received oral anticoagulants at discharge compared to 11.9% in the Canadian study (data about discharge medication were only available in 14.8% of the population in the Canadian study, and no data about discharge medication were available in the US study). Also, in comparison, the two previous studies had even longer follow-up than ours and encountered a higher number of ischemic stroke events post-surgery. Moreover, it has previously been shown that patients post-CABG, irrespectively of presence of AF, have an increased risk for ischemic stroke (25), which to some extent might make it more difficult to show an isolated effect of postoperative AF on the risk of ischemic stroke. Given the current lack of clinical evidence, AF guidelines recommend oral anticoagulants as reasonable for patients with postoperative AF and with risk for ischemic stroke (class IIa, level B recommendation) (26,27). However, this strategy is currently not proven, with only one observational single-center study suggesting that warfarin at discharge might be associated with a lower risk of all-cause mortality (11). Nevertheless, the risk associated with postoperative AF and treatment with oral anticoagulants is an area in need of further research.

### Limitations

This retrospective observational cohort study has some limitations that have to be taken into consideration when interpreting the results. First, patients were not and cannot be randomized, thereby residual confounding may occur. However, we present multicenter data based on all patients undergoing CABG in Sweden with a large sample size and are able to adjust for relevant clinical variables. Furthermore, our findings were confirmed in a sensitivity analysis with propensity-score matching. Still, unknown and unmeasured confounders may exist, and the results should be interpreted with caution. Second, the definition of postoperative AF was based on data entered by physicians on the SWEDEHEART data collection form. However, undetected episodes of AF might be misclassified into the no AF group. Likewise, patients with silent and undiagnosed AF before admission might be misclassified. Third, the definition of AF during follow-up had some inherent limitations. The outcome was based on DC cardioversion as a surrogate marker of symptomatic relapse in AF, probably resulting in us underestimating the relapse risk. However, a similar finding was noted in a sensitivity analysis in which all diagnosis codes of AF during follow-up were accounted for. Unfortunately, this method has its own drawbacks as patients with follow-up visits due to earlier episodes of AF might be wrongly classified as having a relapse in AF, resulting in us overestimating the risk. Despite these limitations, this multicenter cohort of patients after isolated CABG surgery with complete long-term follow-up in routine health care seems to provide valuable information about risks associated with pre- and postoperative AF.

## Conclusions

Among patients undergoing isolated CABG, pre- and postoperative AF was associated with an increased long-term risk of all-cause mortality, cardiovascular mortality, and hospitalization due to congestive heart failure. Moreover, postoperative AF was associated with higher risk of relapse in symptomatic AF. No significant association was observed between AF status and risk for ischemic stroke and myocardial infarction.

## Supplementary Material

Supplemental_material.docx
