# Supplementary material for: Atrial fibrillation in patients undergoing coronary artery surgery is associated with adverse outcome
Source: Ups J Med Sci. 2018 Sep 28;124(1):70–7. doi: 10.1080/03009734.2018.1504148 (PMC6452910; doi:10.1080/03009734.2018.1504148)
Supplement: Supplemental_material.docx [file IUPS_A_1504148_SM9616.docx]

Supplemental online material

Atrial fibrillation in patients undergoing coronary artery surgery
is associated with adverse outcome

Gorav Batra, MD, PhD, Anders Ahlsson, MD, PhD, Bertil Lindahl, MD, PhD,
Lars Lindhagen, PhD, Anders Wickbom, MD, Jonas Oldgren, MD, PhD

**Supplementary Table 1.** Definition of baseline characteristics, in-hospital course, procedures and discharge medication. History of comorbidities was collected from the protocol-standardized SWEDEHEART questionnaires and was enriched with data from the National Patient Registry using the International Classification of Diseases, 9^th^ and 10^th^ revision. Information about drugs at discharge was derived from the National Dispensed Drug Registry using Anatomical Therapeutic Chemical classification codes

| **Variable** | **ICD-code / ATC-code** | **Comments** |
| --- | --- | --- |
| Age | - | Derived from SWEDEHEART |
| Sex | - | Derived from SWEDEHEART |
| Smoking | - | Derived from SWEDEHEART |
| BMI | - | Derived from SWEDEHEART |
| Admission year | - | Derived from SWEDEHEART |
| Atrial fibrillation | I480-I484, I489, 427D | Derived from the National Patient Registry and SWEDEHEART |
| Diabetes mellitus | E10, E11, E12, E13, E14, 250 | Derived from the National Patient Registry and SWEDEHEART based on diabetes/diabetes medication at arrival |
| Hypertension | I10-I15, 401-405 | Derived from the National Patient Registry and SWEDEHEART |
| Myocardial infarction | I21-I23, 410, 412 | Derived from the National Patient Registry and SWEDEHEART |
| Congestive heart failure | I50, I110, I130, I132, K761, P290, 428 | Derived from the National Patient Registry and SWEDEHEART |
| Peripheral vascular disease | I70-I73, 440-443 | Derived from the National Patient Registry |
| Thromboembolism | I74, 444 | Derived from the National Patient Registry |
| Ischemic stroke | I63, 433, 434 | Derived from the National Patient Registry |
| Unknown stroke | I64 | Derived from the National Patient Registry |
| Transient ischemic attack | G45, 435 | Derived from the National Patient Registry |
| Hemorrhagic stroke | I60, I61, 430, 431 | Derived from the National Patient Registry |
| Any bleeding | I850, K226, K250, K252, K254, K256, K260, K262, K264, K266, K270, K272, K274, K276, K280, K284, K286, K290, K625, K661, K920- K922, I60-I62, N02, R310, R311, R318, R040-R042, R048, R049, R58, T810, I983, D629, DR029 | Derived from the National Patient Registry |
| Renal failure | N17, N18, N19, N990, O084, I120, I131, I132, T795, P960, Z49, V56A, V45B, Z992, 584, 585, 586 | Derived from the National Patient Registry |
| Chronic obstructive pulmonary disease | J40-J47, 49-496 | Derived from the National Patient Registry |
| Dementia | G30, G31, 290, 294B | Derived from the National Patient Registry |
| Cancer diagnosis within 3 years | C14-C20 | Derived from the National Patient Registry |
| Previous PCI | FNG00, FNG01, FNG02, FNG03, FNG04, FNG05, FNG06, 3080 | Derived from the National Patient Registry and SWEDEHEART |
| Previous CABG | FNA-FNF, FNH-FNW, 3065, 3066, 3068, 3092, 3105, 3127, 3158 | Derived from the National Patient Registry and SWEDEHEART |
| Indication for CABG | - | Derived from SWEDEHEART |
| EuroSCORE I | - | Derived from SWEDEHEART |
| Extracorporeal circulation | - | Derived from SWEDEHEART |
| Number of central anastomoses | - | Derived from SWEDEHEART |
| Number of peripheral anastomoses | - | Derived from SWEDEHEART |
| Internal mammary artery, left | - | Derived from SWEDEHEART |
| Internal mammary artery, right | - | Derived from SWEDEHEART |
| Vein graft | - | Derived from SWEDEHEART |
| Radial artery graft | - | Derived from SWEDEHEART |
| Postoperative atrial fibrillation | - | Derived from SWEDEHEART |
| Postoperative stroke | - | Derived from SWEDEHEART |
| Postoperative bleeds | - | Derived from SWEDEHEART |
| Left ventricular ejection fraction | - | Derived from SWEDEHEART |
| Creatinine | - | Derived from SWEDEHEART |
| Aspirin | B01AC06 | Derived from the National Dispensed Drug Registry |
| P2Y_12_ inhibitors | B01AC04, B01AC22, B01AC24 | Derived from the National Dispensed Drug Registry |
| Oral anticoagulants | B01AA03, B01AE07, B01AF01, B01AF02 | Derived from the National Dispensed Drug Registry |
| ACEI/ARB | C09A, C09B, C09C, C09D | Derived from the National Dispensed Drug Registry |
| Calcium channel blockers | C08 | Derived from the National Dispensed Drug Registry |
| Diuretics | C03 | Derived from the National Dispensed Drug Registry |
| Lipid lowering agents | C10 | Derived from the National Dispensed Drug Registry |
| Digoxin | C01AA05 | Derived from the National Dispensed Drug Registry |
| β-blockers | C07 | Derived from the National Dispensed Drug Registry |
| Sotalol | C07AA07, C07BA07, C07AA57 | Derived from the National Dispensed Drug Registry |
| Amiodarone | C01BD01 | Derived from the National Dispensed Drug Registry |
| Verapamil / Diltiazem | C05AE03, C08DA01, C08DA51, C08DB01, C09BB10 | Derived from the National Dispensed Drug Registry |

**Supplementary Table 2.** Definition of outcomes. The National Patient Registry, the Swedish Cause of Death Registry and the International Classification of Diseases, 10^th^ revision, was used to define the outcomes

| **Variable** | **ICD-code** | **Comments** |
| --- | --- | --- |
| All-cause mortality | - | Derived from the Swedish Cause of Death Registry |
| Cardiovascular mortality | I00-I99 | Derived from the Swedish Cause of Death Registry |
| Myocardial infarction | I21, I22 | Derived from the National Patient Registry and the Swedish Cause of Death Registry |
| Congestive heart failure | I50, I110, I130, I132, K761, P290 | Derived from the National Patient Registry and the Swedish Cause of Death Registry |
| Ischemic stroke | I63 | Derived from the National Patient Registry and the Swedish Cause of Death Registry |
| Recurrent symptomatic atrial fibrillation (DC cardioversion) | DF010, DF026, DF027 | Derived from the National Patient Registry |
| Recurrent atrial fibrillation (diagnosis) | I480-I484, I489 | Derived from the National Patient Registry and the Swedish Cause of Death Registry |

**Supplementary Table 3.** Propensity score hazard ratios with a 95% confidence interval for postoperative atrial fibrillation versus no atrial fibrillation

| **Outcome** | **Hazard ratio (95% confidence interval)** |
| --- | --- |
| All-cause mortality | 1.35 (1.02 – 1.79) |
| Cardiovascular mortality | 1.78 (1.17 – 2.73) |
| Myocardial infarction | 1.29 (0.93 – 1.80) |
| Congestive heart failure | 1.40 (1.08 – 1.81) |
| Ischemic stroke | 1.12 (0.76 – 1.64) |
| Recurrent AF | 4.20 (1.84 – 9.62) |
